# Oxidative Stress as Estimated by Gamma-Glutamyl Transferase Levels Amplifies the Alkaline Phosphatase-Dependent Risk for Mortality in ESKD Patients on Dialysis

**DOI:** 10.1155/2016/8490643

**Published:** 2016-07-25

**Authors:** Claudia Torino, Francesco Mattace-Raso, Jan L. C. M. van Saase, Maurizio Postorino, Giovanni Luigi Tripepi, Francesca Mallamaci, Carmine Zoccali, PROGREDIRE Study Group

**Affiliations:** ^1^CNR-IFC, Clinical Epidemiology and Physiopathology of Renal Diseases and Hypertension of Reggio Calabria, 89124 Reggio Calabria, Italy; ^2^Department of Internal Medicine, Erasmus University Medical Centre, 3015 CE Rotterdam, Netherlands; ^3^CNR-IFC and Nephrology, Dialysis and Transplantation Unit, Ospedali Riuniti, c/o EUROLINE S.r.l., Via Vallone Petrara 57-59, 89124 Reggio Calabria, Italy

## Abstract

Alkaline phosphatase (Alk-Phos) is a powerful predictor of death in patients with end-stage kidney disease (ESKD) and oxidative stress is a strong inducer of Alk-Phos in various tissues. We tested the hypothesis that oxidative stress, as estimated by a robust marker of systemic oxidative stress like *γ*-Glutamyl-Transpeptidase (GGT) levels, may interact with Alk-Phos in the high risk of death in a cohort of 993 ESKD patients maintained on chronic dialysis. In fully adjusted analyses the HR for mortality associated with Alk-Phos (50 IU/L increase) was progressively higher across GGT quintiles, being minimal in patients in the first quintile (HR: 0.89, 95% CI: 0.77–1.03) and highest in the GGT fifth quintile (HR: 1.13, 95% CI: 1.03–1.2) (*P* for the effect modification = 0.02). These findings were fully confirmed in sensitivity analyses excluding patients with preexisting liver disease, excessive alcohol intake, or altered liver disease biomarkers. GGT amplifies the risk of death associated with high Alk-Phos levels in ESKD patients. This observation is compatible with the hypothesis that oxidative stress is a strong modifier of the adverse biological effects of high Alk-Phos in this population.

## 1. Introduction

Tissue nonspecific alkaline phosphatase (Alk-Phos) is an enzyme highly represented in the bone and in the liver and the measurement of the activity of this enzyme is a time-honored biomarker applied for the diagnosis and the clinical monitoring of bone and liver diseases [1]. Alk-Phos catalyzes the hydrolysis of pyrophosphate, the main calcification inhibitor, and seminal studies in patients with end-stage kidney disease (ESKD) documented that circulating Alk-Phos activity is robustly related to the risk of death [2–4]. In ESKD patients Alk-Phos mainly reflects increased bone turnover [1], triggered and maintained by secondary hyperparathyroidism and modulated by several other factors among which oxidative stress [5] plays a relevant role. Oxidative stress is notoriously pervasive in ESKD patients [6, 7]. Among biomarkers of oxidative stress, *γ*-Glutamyl-Transpeptidase (GGT) is now regarded as one of the most robust indicators of whole body oxidative stress [8, 9]. High levels of GGT predict mortality in ESKD patients [10, 11] and in the general population [12] being associated with a high risk for coronary heart disease [12, 13] and heart failure [14]. Of note, oxidative stress is a powerful inducer of Alk-Phos in vascular and bone cells [15] and is key to vascular calcification [16]. Even though the predictive power of Alk-Phos for adverse clinical outcomes has been previously confirmed in ESKD [17–23], the possible interaction between Alk-Phos and biomarkers of oxidative stress like GGT has not been investigated so far. As oxidative stress and mineral metabolism are intimately related phenomena in ESKD [5], we investigated if GGT modifies the association between Alk-Phos and all-cause and cardiac mortality in a sizable cohort of patients with ESKD maintained on chronic dialysis.

## 2. Methods

The study protocol was approved by the ethical committee of our institution. All participants gave their informed consent before enrolment.

### 2.1. Study Population

The study population is part of a cohort of 1189 dialysis patients enrolled in the PROGREDIRE (Prospective Registry of The Working Group of Epidemiology of Dialysis Region Calabria), a cohort study involving 35 dialysis units in two regions in Southern Italy (Calabria and Sicily). We included in this analysis 993 patients in which both Alk-Phos and GGT measurements were available. Patients where Alk-Phos and GGT were not available (*n* = 196, 16%) did not differ from those included in the study for any of the main demographic, clinical, and biochemical characteristics listed in Table 1.

Patients had been on regular dialysis [haemodialysis (HD) or peritoneal dialysis (PD)] for a median time of 3.0 years (interquartile range: 1.8–4.4 years). HD patients (*n* = 932) were being treated with standard bicarbonate dialysis with noncellulosic membrane filters of various types. PD patients (*n* = 61) were either on 4 standard exchanges per day or on continuous cycling peritoneal dialysis. Six hundred and thirty-four patients were treated with various antihypertensive drugs (271 on monotherapy with ACE inhibitors, calcium channel blockers, *α*- and *β*-blockers, vasodilators, diuretics, or other drugs, 194 on double therapy, 92 on triple therapy, and 77 patients on quadruple or quintuple therapy with various combinations of these drugs). The main demographic, somatometric, clinical, and biochemical characteristics of the study population are detailed in Table 1.

### 2.2. Laboratory Measurements

Blood sampling was performed at baseline after an overnight fast. For HD patients, blood was always drawn during a mid-week day (brief dialysis interval). Alk-Phos, GGT, cholesterol, albumin, calcium, phosphate, C-Reactive Protein (CRP), haemoglobin, Glutamic-Oxaloacetic Transaminase (GOT), and Glutamic-Pyruvic Transaminase (GPT) measurements were made using standard methods in the routine clinical laboratory. In our laboratory, the normal range of Alk-Phos was 30 to 120 UI/L and that of GGT was 0–45 UI/L.

### 2.3. Study End Points

Mortality and fatal and nonfatal cardiac events were the main study end points. Cardiac events were classified as follows: myocardial infarction confirmed by serial changes of ECG and cardiac biomarkers; ECG-documented angina episodes; ECG-documented arrhythmia; unexpected, sudden death highly suspected as of cardiac origin. De novo chronic heart failure (CHF) was defined as CHF in a patient without CHF at baseline. To be classified as having CHF patients had to show mild or more severe dyspnoea during ordinary activities (NYHA class II or higher) plus evidence of anatomical/functional LV disease on echocardiography. Each cause of death was assessed by 3 independent physicians. In doubtful cases, diagnosis was attributed by consensus. During the review process, involved physician used all available medical information, including hospitalization forms and medical records. In case of death occurring at home, family members and/or general practitioners were interviewed to better understand the circumstances which led to death.

### 2.4. Statistical Analysis

Data were expressed as mean ± standard deviation (normally distributed data) or median and interquartile range (nonnormally distributed data) or as percent frequency (categorical data). Comparisons among groups were made by one-way ANOVA, Kruskal-Wallis, or Chi Square test, as appropriate. Regression analysis was performed to investigate the relationship between Alk-Phos, GGT, and markers of liver function and bone mineral metabolism. Due to the nonnormal distribution of both Alk-Phos and GGT both variables were log-transformed before analysis. Survival analyses were performed by using both univariate and multivariate Cox regression analyses, including Alk-Phos, GGT, and their interaction term as well as traditional [age, gender, current smoking, diabetes, cholesterol, arterial pressure and antihypertensive treatment, and cardiovascular comorbidities], inflammation and nutritional status [CRP, BMI, and albumin], and ESKD-related risk factors [dialysis vintage, haemoglobin]. ALT, AST, HbsAg, HCV, alcohol consumption, and preexisting liver disease were always included in the multivariate models. The hazard ratios of alkaline phosphatase across GGT categories were calculated by the standard linear combination method. The best functional form of GGT (i.e., quintiles) was chosen by analysing the Martingale residuals in Cox's regression analysis [24]. Multivariate models were built as previously described. Statistical analysis was performed by using standard statistical packages (SPSS for Windows, Version 20, Chicago, Illinois, USA; STATA for Windows, Version 13, College Station, Texas, USA).

## 3. Results

**Figure 1:**
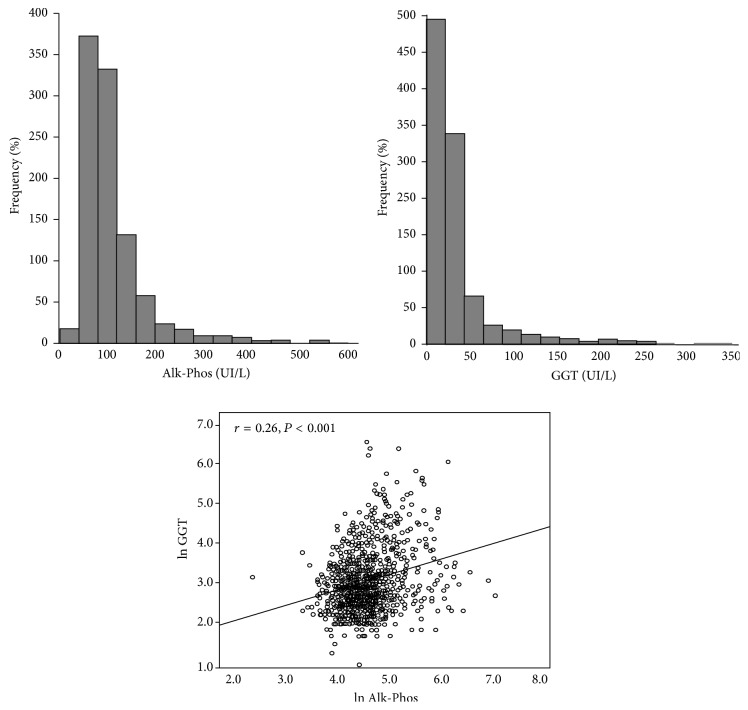
Distribution of Alk-Phos and GGT and their correlation in the study population.

The main baseline characteristics of the study population are reported in Table 1. Both Alk-Phos and GGT distributions were right-skewed and the median value of the two biomarkers was 89 UI/L and 20 UI/L, respectively (Figure 1). Two hundred and seventy-one patients (27%) had Alk-Phos exceeding the upper limit of the normal range of this biomarker (120 UI/L) and 83 (17%) had GGT greater than 45 UI/L (the upper limit of the normal range). Sixty-three percent of patients were males and mean age was 65 years. Diabetics were 28%. Alk-Phos levels were higher in female patients (median 97 UI/L, IQR: 74–140 UI/L) than in male patients (median 85 UI/L, IQR: 64–116 UI/L). Patients with higher levels of Alk-Phos had been on dialysis for longer time and had higher CRP levels. Conversely, calcium and phosphate levels showed an opposite trend (Table 1).

### 3.1. Correlates of Alkaline Phosphatase and *γ*-Glutamyl-Transpeptidase

Alk-Phos showed a direct, highly significant association with GGT (*r* = 0.26, *P* < 0.001) (Figure 1). Furthermore, Alk-Phos was directly associated with GOT (*r* = 0.13, *P* < 0.001), GPT (*r* = 0.14, *P* < 0.001), and Parathyroid Hormone (PTH) (*r* = 0.38, *P* < 0.001) and correlated inversely with calcium (*r* = −0.13, *P* < 0.001) and phosphate (*r* = −0.16, *P* < 0.001). The same variables, except PTH, were associated with GGT [GGT versus GOT (*r* = 0.40, *P* < 0.001); GGT versus GPT (*r* = 0.41, *P* < 0.001); GGT versus calcium (*r* = −0.08, *P* = 0.01); GGT versus phosphate (*r* = −0.14, *P* < 0.001)].

### 3.2. Survival Analysis: All-Cause Death

**Figure 2:**
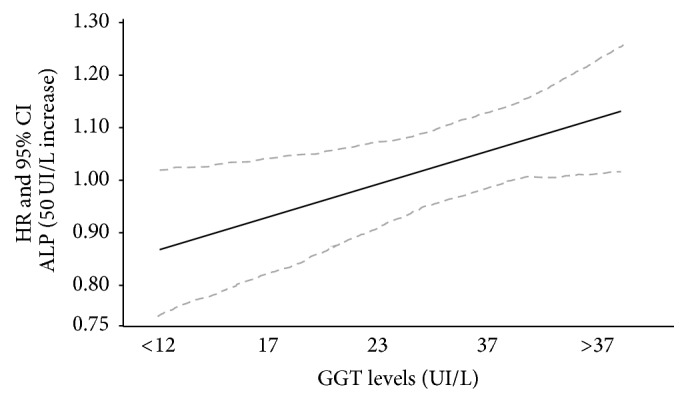
Effect modification by *γ*-Glutamyl-Transpeptidase on the relationship between alkaline phosphatase and all-cause mortality. The HR in this graph represents the risk for all-cause death due to alkaline phosphatase across *γ*-Glutamyl-Transpeptidase levels.

During a median follow-up of 3.0 years (interquartile range: 1.8–4.4 years), 405 patients died. In a basic model including Alk-Phos, GGT, and their interaction term, GGT significantly amplified the risk of death across progressively increasing Alk-Phos levels (*P* for the effect modification = 0.004) (Table 2, crude analysis). These results were confirmed in fully adjusted analyses, where the risk associated with 50 UI/L increase of in Alk-Phos for all-cause mortality was progressively higher from the first to the fifth quintile (1st quintile: HR: 0.89, 95% CI: 0.77–1.03; 2nd quintile: HR: 0.95, 95% CI: 0.85–1.05; 3rd quintile: HR: 1.01, 95% CI: 0.94–1.08; 4th quintile HR: 1.07, 95% CI: 1.01–1.14, 5th quintile HR: 1.13, 95% CI: 1.03–1.2) (*P* for the effect modification = 0.02). (Table 2; Figure 2). Exclusion of heavy drinkers (*n* = 23) and of patients affected by chronic liver diseases (*n* = 68) only modestly reduced the HR of the Alk-Phos-GGT interaction (HR: 1.06, 95% CI: 1.01–1.12).

## 4. Discussion

In this study, GGT, a systemic marker of oxidative stress, emerged as a coherent amplifier of the death risk portended by high Alk-Phos in ESKD patients on dialysis. This interaction was largely independent of liver disease and alcohol intake and was confirmed in sensitivity analyses excluding patients with preexisting liver disease or self-reported high alcohol intake. Overall, these findings suggest that systemic oxidative stress, as estimated by GGT, plays a relevant role in predicting the risk for major clinical outcomes portended by increased alkaline phosphatase.

Alk-Phos is an established predictor of death in ESKD patients on haemodialysis. Several studies reported a linear association between Alk-Phos levels and mortality in ESKD [2–4, 17–23]. Additional studies focusing on predialysis CKD patients showed that such a link is not peculiar to the end-stage phase of CKD [25–27]. Furthermore, observational studies in various communities documented that Alk-Phos is a quite strong risk factor for death and cardiovascular events in the general population [28]. This enzyme is ubiquitous and located at cell surface and it is directly involved in glutathione catabolism, the main antioxidant system in humans [29, 30]. Circulating levels of Alk-Phos in ESKD in patients without obvious liver disease mainly reflect bone turnover [31]. In this regard, it is worth mentioning that, in in vitro experiments in vascular and bone cells, oxidative stress is a strong inducer of alkaline phosphatase and a key event promoting the transition of the vascular cells phenotype into calcifying cells [15]. Alk-Phos is seen as a host defence molecule that is part of the innate immune response to bacterial agents. Indeed this enzyme is potently induced by IL-6, TNF-*α*, and bacterial lipopolysaccharide, all factors typically associated with inflammation and high oxidative stress [32]. Thus the hypothesis that oxidative stress may interact with Alk-Phos in organ damage and ultimately in major clinical outcomes is biologically well founded. However, in no study was such an interaction formally investigated. The issue is of particular relevance in ESKD because high levels of oxidative stress are a hallmark in these patients [6, 7]. Various biomarkers of oxidative stress are currently applied in clinical research [33–36] and among these GGT is seen as the one that better captures whole body oxidative stress [8]. Of note, in previous studies in ESKD, GGT exhibited a much stronger link with mortality [10] than other oxidative stress biomarkers tested in the same population. With this background in mind we set out to make a detailed analysis of the interaction between GGT and alkaline phosphatase for risk of death, the most solid outcome measure in clinical studies. Along with the working hypothesis we found that GGT is a relevant modifier of the risk of Alk-Phos for mortality. Indeed in a crude analysis the interaction term indicated that a fixed increase in Alk-Phos levels (50 UI/L) produced a stepwise increase in the risk of death across GGT quintile (HR = 1.08 per GGT quintile). Importantly, this interaction was unmodified by adjustment for a comprehensive series of risk factors for mortality, including CV comorbidities, clinical risk factors, BMI, risk factors peculiar to ESKD like haemoglobin, C-reactive protein, serum albumin and phosphate, and biomarkers of liver disease and alcohol intake. The fact that the interaction was independent of concomitant liver disease indicates that the effect of modification of GGT on the Alk-Phos death relationship does not represent a mere effect of liver damage but a more general phenomenon, more likely oxidative stress [8].

Our study has limitations. First, our observations are limited to a single ESKD cohort of Caucasian patients. Therefore, confirmation in a second ESKD cohort and in studies in other ethnicities is still needed for establishing the external generalizability of our findings. Second, even though we did a comprehensive adjustment for a long list of potential confounders, confounding for unmeasured and/or unknown risk factors remains possible, an issue that can be solved only by a clinical trial. In this regard, it is worth mentioning that drugs that reduce serum Alk-Phos are being investigated and that one of these drugs has produced a significant reduction in vascular calcification and bone loss in ESKD patients [37]. Our findings suggest that the protective effect of this drug may be attenuated in patients with high GGT, hypothesis which may be formally tested in secondary analyses of the same trial.

## Figures and Tables

**Table 1:** Main demographic, somatometric and clinical characteristics in the whole study population and in patients as divided according to alkaline phosphatase quartiles.

	Whole group (*n* = 993)	Alk-Phos < median value (*n* = 497)	Alk-Phos > median value (*n* = 496)	*P* for linear trend
Age (years)	65 ± 14	65 ± 14	65 ± 13	0.93
BMI (kg/m^2^)	25 ± 5	25 ± 5	25 ± 5	0.74
Male sex *n*. (%)	624 (63)	343 (69)	281 (57)	**<0.001**
Current smokers *n*. (%)	149 (15)	78 (16)	71 (14)	0.54
Past smokers *n*. (%)	370 (37)	202 (41)	168 (34)	**0.03**
Diabetics *n*. (%)	272 (28)	127 (26)	145 (30)	0.14
On antihypertensive treatment *n*. (%)	634 (64)	320 (64)	314 (63)	0.72
Dialysis vintage (months)	45 (21–85)	38 (19–76)	52 (26–96)	**<0.001**
With cardiovascular comorbidities^*∗*^ *n*. (%)	533 (54)	257 (52)	276 (56)	0.21

Systolic blood pressure (mmHg)	135 ± 22	135 ± 22	135 ± 23	0.99
Diastolic blood pressure (mmHg)	74 ± 12	74 ± 12	73 ± 11	0.09
Pulse pressure (mmHg)	74 ± 11	73 ± 10	74 ± 11	0.13

Cholesterol (mg/dL)	156 ± 40	155 ± 39	156 ± 41	0.61
Haemoglobin (g/dL)	11.3 ± 1.5	11.3 ± 1.4	11.3 ± 1.5	0.96
Albumin (g/dL)	3.9 ± 0.5	3.9 ± 0.5	3.9 ± 0.5	0.87
CRP (mg/L)	5.0 (3.0–13.0)	4.1 (2.9–12.0)	5.7 (3.0–14.0)	**0.02**
Calcium (mg/dL)	9.1 ± 0.9	9.2 ± 0.9	9.0 ± 0.9	**0.001**
Phosphate (mg/dL)	5.0 ± 1.6	5.2 ± 1.6	4.9 ± 1.6	**0.001**

^*∗*^Cardiovascular comorbidities: the presence, at baseline, of at least one of these comorbidities: angina, arrhythmia, myocardial infarction, coronary surgery, angioplasty, other heart surgeries, claudicatio intermittens, amputations, peripheral surgery, stroke, TIA, and preexisting chronic heart failure.

Data are expressed as mean ± SD or median and interquartile range or as percent frequency, as appropriate.

**Table 2:** Crude and adjusted Cox regression analyses showing the effect modification of *γ*-Glutamyl-Transpeptidase on alkaline phosphatase for all-cause mortality. The criteria for building these models are detailed in the methods.

Variables (units of increase)	Crude analysis	Fully adjusted analysis
Alk-Phos (50 UI/L)	0.80 (0.67–0.96), *P* = 0.02	0.84 (0.69–1.02), *P* = 0.08
GGT (quintiles)	0.96 (0.84–1.09), *P* = 0.52	0.97 (0.85–1.12), *P* = 0.72
Alk-Phos*∗*GGT (50 UI/L*∗*quintiles)	1.08 (1.02–1.13), *P* = 0.004	1.06 (1.01–1.12), *P* = 0.02
Age (1 year)		1.05 (1.04–1.06), *P* < 0.001
Gender (0 = female; 1 = male)		0.97 (0.77–1.21), *P* = 0.77
Current smoking (0 = no; 1 = yes)		0.93 (0.67–1.29), *P* = 0.66
Diabetes (0 = no; 1 = yes)		1.29 (1.03–1.62), *P* = 0.03
Systolic blood pressure (1 mmHg)		1.00 (0.99–1.00), *P* = 0.67
CV comorbidities^a^ (0 = no; 1 = yes)		1.55 (1.24–1.94), *P* < 0.001
Antihypertensive treatment (0 = no; 1 = yes)		1.12 (0.90–1.39), *P* = 0.98
Dialysis vintage (1 month)		1.00 (1.00-1.00), *P* < 0.001
Cholesterol (1 mg/dL)		1.00 (1.00-1.00), *P* = 0.002
Hb (1 g/dL)		0.93 (0.86–0.99), *P* = 0.04
Phosphate (1 mg/dL)		1.01 (0.94–1.08), *P* = 0.80
Albumin (1 g/dL)		0.70 (0.55–0.88), *P* = 0.002
CRP (1 mg/L)		1.00 (1.00-1.00), *P* = 0.56
Body Mass Index (BMI) (1 kg/m^2^)		0.99 (0.97–1.02), *P* = 0.46
GOT (1 UI/L)		1.01 (0.99–1.03), *P* = 0.45
GPT (1 UI/L)		1.00 (0.99–1.01), *P* = 0.98
Bilirubin (1 mg/dL)		0.91 (0.54–1.51), *P* = 0.70
HbsAg (0 = no; 1 = yes)		0.73 (0.37–1.44), *P* = 0.37
HCV (0 = no; 1 = yes)		0.81 (0.56–1.16), *P* = 0.25
Cirrhosis/hepatitis (0 = no; 1 = yes)		1.26 (0.71–2.24), *P* = 0.44
Current alcohol consumption (0 = no; 1 = yes)		1.12 (0.88–1.42), *P* = 0.34

Data are expressed as hazard ratio, 95% confidence interval (CI), and *P* values.

^a^CV comorbidities were defined as in Table 1.
